# Sex‐Specific Effects of APOE Genotype on Contextual Fear Discrimination

**DOI:** 10.1002/alz.095544

**Published:** 2025-01-09

**Authors:** Hassan E Mohammed, Jackie Coniglio, Victor M. Luna

**Affiliations:** ^1^ Temple University, Philadelphia, PA USA

## Abstract

**Background:**

The Apolipoprotein E4 (APOE4) allele is the strongest genetic risk factor for sporadic late‐onset Alzheimer’s disease (AD). Moreover, the link between APOE4 and AD is more pronounced in women than in men. In this study, we investigate how APOE genotype and sex impact emotional pattern separation. This is a cognitive faculty that enables discrimination between highly similar emotional and non‐emotional experiences, deficits of which are key neuropsychiatric symptoms of AD progression.

**Method:**

To determine the role of APOE alleles in emotional pattern separation, 6‐month‐old male (n = 14) and female mice (n = 15) expressing humanized APOE2, 3 or 4 alleles (CureAlz) were tested on a contextual fear discrimination (CFD) task that is dependent on the dentate gyrus and on adult hippocampal neurogenesis. Mice were evaluated on their ability to distinguish between an aversive Context A (with foot shock) and a similar yet non‐aversive Context B (no foot shock) for 5 days. For each Context, visual and olfactory stimuli in the chamber were altered while the tactile features of the floor were kept the same. As part of CFD protocol, mice also underwent standard contextual fear conditioning (CFC) testing.

**Result:**

We found no significant effect of sex or APOE allele on CFC learning. However, in the CFD task, we found that female E2 mice performed significantly better than female E3 and E4 animals. E3 females appeared to discriminate between aversive and non‐aversive contexts better than E4. In contrast, we found that APOE allele did not impact CFD in males. Finally, we found that E2 females significantly outperformed E2 males in the CFD task. E3 and E4 females performed similar to their male counterparts.

**Conclusion:**

Our results suggest that early in adulthood, the APOE2 allele greatly impacts emotional pattern separation in a sex‐dependent manner. To determine the underlying synaptic mechanisms for our CFD findings, we have been investigating how APOE alleles impact the expression of all glutamate receptor subunits in the dentate gyrus as well as neurogenesis levels. Most importantly, we have been aging our mouse colonies to determine how age, APOE allele, sex, and glutamate receptor composition together dictate emotional pattern separation related to AD.

